# Concerns of older patients and their caregivers in the emergency department

**DOI:** 10.1371/journal.pone.0235708

**Published:** 2020-07-09

**Authors:** Noortje Zelis, Sarah E. Huisman, Arisja N. Mauritz, Jacqueline Buijs, Peter W. de Leeuw, Patricia M. Stassen

**Affiliations:** 1 Department of Internal Medicine and Gastroenterology, Zuyderland Medical Center, Heerlen, The Netherlands; 2 Division of General Internal Medicine, Department of Internal Medicine, Section Acute Medicine, Maastricht University Medical Center, Maastricht University, Maastricht, The Netherlands; 3 School of CAPHRI, Maastricht University Medical Center, Maastricht University, Maastricht, The Netherlands; 4 CARIM School for Cardiovascular Diseases, Maastricht University Medical Center, Maastricht University, Maastricht, The Netherlands; Wayne State University, UNITED STATES

## Abstract

**Background:**

Older emergency department (ED) patients often have complex problems and severe illnesses with a high risk of adverse outcomes. It is likely that these older patients are troubled with concerns, which might reflect their preferences and needs concerning medical care. However, data regarding this topic are lacking.

**Methods:**

This study is a sub study of a prospective, multicenter, observational cohort study among older medical ED patients (≥65 years). Patients or their caregivers were asked about their illness-related concerns during the first stage of the ED visit using a questionnaire. All concerns were categorized into 10 categories, and differences between patients and caregivers, and between age groups were analyzed. Odds Ratios were calculated to determine the association of the concerns for different adverse outcomes.

**Results:**

Most of the 594 included patients (or their caregivers) were concerned (88%) about some aspects of their illness or their need for medical care. The most often reported concerns were about the severity of disease (43.6%), functional decline (9.4%) and dying (5.6%). Caregivers were more frequently concerned than patients (p<0.001) especially regarding the severity of disease (50.5 vs 39.6%, p = 0.016) and cognitive decline (10.8 vs. 0.3%, p <0.001). We found no difference between age groups. The concern about dying was associated with 30-day mortality (OR 2.89; 95%CI: 1.24–6.70) and the composite endpoint (intensive- or medium care admission, length of hospital stay >7 days, loss of independent living and unplanned readmission within 30 days) (OR 2.32; 95%CI: 1.12–4.82). In addition, unspecified concerns were associated with mortality (OR 1.88; 95%CI: 1.09–3.22).

**Conclusion:**

The majority of older patients and especially their caregivers are concerned about their medical condition or need for medical care when they visit the ED. These concerns are associated with adverse outcomes and most likely reflect their needs regarding medical care. More attention should be paid to these concerns because they may offer opportunities to reduce anxiety and provide care that is adjusted to their needs.

**Trial registration:**

This study was registered on clinicalTriagls.gov (NCT02946398).

## Introduction

In all likelihood, older patients (≥65 years) who have been referred to an emergency department (ED) experience great concerns regarding their condition and their need for medical care. This is not surprising as these patients often present with complex problems and severe illnesses with a high risk of an adverse outcome, including the loss of independence and death [1–3].

Nevertheless, only a few studies have reported on the concerns of ED patients [4–7], but these focused mainly on their overall experience during the ED visit and not explicitly on their concerns. In addition, these studies were performed in younger patients and/or in small populations [5, 6, 8, 9]. Only in one study, patients were explicitly asked about their concerns, but this was done in an out-patient setting [6]. Since many patients feel inhibited to talk about their feelings to the ED staff [8, 10], it is likely that their concerns have not been completely exposed in these studies. Recently, we performed the RISE UP study to find predictors of adverse outcomes in older ED patients [11], and found that the severity of concerns in patients and caregivers is predictive of adverse outcomes [12]. It may, therefore, also be possible that specific concerns are predictive of that particular outcome.

We specifically explored the nature of the concerns and their prognostic value in the RISE UP study cohort consisting of older ED patients. We hypothesized that older ED patients or their caregivers experience a broad spectrum of concerns regarding their medical condition and medical care, which may be associated with adverse clinical outcomes. We further focused on the types of concerns of patients, caregivers and different age groups.

## Materials and methods

### Study design, setting and study population

This study is part of the RISE UP study, a prospective multicenter observational cohort study, aiming to identify predictors for adverse outcome in older medical ED patients. In short, this study was conducted at the EDs in Zuyderland Medical Center (MC) and Maastricht University Medical Center+ (MUMC+) in The Netherlands from July 2016 until February 2017. Older ED patients (≥65 years) treated by internists or gastroenterologists were eligible for inclusion after informed consent was obtained from the patient or legal representative. The study protocol was approved by the medical ethics committees of Zuyderland MC and MUMC+ (NL55867.096.15) and published online [11]. This study was reported in line with the STROBE (STrengthening the Reporting of OBservational studies in Epidemiology) guidelines [13].

### Data collection

In the RISE UP study, all participants (patients or caregivers) received a questionnaire, which was filled out immediately after admission to the ED, before history taking and physical examination by the physician. The questionnaire contained questions about their concerns, disease perception and self-rated health. Results of this questionnaire, regarding the predictive value of clinical intuition for adverse outcomes, have been published online [12]. For the current study, we focused on the categorical question: ‘Are you concerned about your (his/her) condition?’ and the open question ‘If you are concerned about your (his/her) condition, what are you concerned about?’. All the answers to the categorical and open questions were entered in SPSS. Subsequently, all answers were categorized independently by two researchers into the following ten categories: no concern; severity of disease; functional decline; cognitive decline; dying; relatives; diagnostic procedures or treatment; not being acknowledged; miscellaneous and not further specified. As some patients/caregivers mentioned more than one illness-related concern, we included and analyzed the two first mentioned concerns. In case of disagreement between the two researchers, a third researcher decided on the issue.

Additionally, we retrieved from the medical records the following data to phenotype our population: demographics, living situation, comorbidities (quantified according to the Charlson Comorbidity Index (CCI) [14]) and cognitive function. Functional status was assessed using a questionnaire to determine the Katz Activities of Daily Living (ADL) index score [15], which was filled out for all hospitalized patients. The main reason for the ED-visit was categorized according to the International Classification of Diseases (ICD)-10 [16].

### Outcome measures

The primary outcome of this study was 30-day all-cause mortality. The secondary endpoints were a) a composite endpoint consisting of intensive or medium care unit (ICU/MCU) admission, prolonged length of hospital stay (LOS; >7 days), loss of independent living and unplanned readmission within 30 days after discharge, and b) loss of independent living. All patients were followed up for at least 30 days to obtain outcomes.

To assess the association of the illness-related concern categories “no concern”, “severity of disease”, “dying” and “not further specified” with adverse outcomes, we used 30-day mortality and the secondary composite outcome. For the two concern categories “functional and cognitive decline”, we used the secondary loss of independent living outcome. We chose to evaluate the association between these six concern categories and adverse outcome because we hypothesized that these specific categories would be associated with the outcome measures.

### Statistical analysis

The sample size of this study was based on that of the RISE UP study. We performed descriptive analyses of the baseline characteristics, types of concerns and outcome measures. For the analysis regarding differences of concerns between patients and caregivers, we excluded questionnaires in which it was unclear whether the patient or the caregiver filled out the form. To study the differences in concerns between age groups, the study population was divided into two groups: 65–79 years and ≥80 years of age. Differences in concerns between patients and caregivers and between age groups were analyzed using the Chi-square or Fisher’s Exact tests, when appropriate. We used univariable logistic regression analyses to determine the predictive value of specific concerns for the outcomes. Odds ratios (ORs) with 95% confidence intervals (CIs) were calculated. All analyses were performed using IBM SPSS Statistics for Windows, Version 24.0 (IBM Corp., Armonk, N.Y., USA). P-values <0.05 were considered statistically significant.

## Results

### Study population and characteristics

During the study period 603 patients were included. Detailed information regarding the patient selection was described in our previous article [12]. Questionnaires were missing in 9 patients, so 594 patients were included in the final analysis. The median age of the patients was 79 years (IQR 73–85) and 52% were male (Table 1). Most patients were community-dwelling (86.7%) with a median Katz-ADL score of 0, which means that most of the patients were independent with respect to ADL. Cognitive impairment (including delirium) was present in 164 (29.0%) patients.

**Table 1:** Patient characteristics.

	All patients N = 594
Demographics	
Median (IQR) Age, years	79 (73–85)
Male, n, %	309 (52.0)
Community dwelling, n (%)	515 (86.7)
Comorbidity and functional status	
Median (IQR) CCI score	2 (1–3)
Median (IQR) Katz-ADL index score ^a^	0 (0–2)
Dementia, mild cognitive impairment or delirium, n (%)^b^	164 (29.0)
Reason for ED-visit (ICD-10), n (%)	
Infectious and parasitic disease	174 (29.3)
Diseases of the digestive system	155 (26.1)
Diseases of the circulatory system	54 (9.1)
Neoplasms	50 (8.4)
Endocrine, nutritional and metabolic diseases	31 (5.2)
Diseases of the respiratory system	30 (5.1)
Diseases of blood and blood-forming organs	27 (4.5)
Diseases of the genitourinary system	27 (4.5)
Miscellaneous	46 (7.7)

SD, Standard Deviation; CCI, Charlson Comorbidity Index; ADL, Activities of Daily Living; ICD-10, International Classification of Diseases-10

^a^Katz-ADL index score determined in all hospitalized patients (n = 472).

^b^Denominator: n = 566

### Outcomes

Of the 594 patients, 64 (10.8%) died within 30 days after the ED visit (primary outcome). In total, 262 (44.1%) met the secondary composite outcome (ICU/MCU admission, prolonged LOS, loss of independent living and unplanned readmission within 30 days after discharge). The secondary loss of independent living outcome occurred in 82 (14.8%) patients. Follow up was complete for all patients with regard to all outcome measures.

### Concerns of patients and caregivers

**Fig 1:**
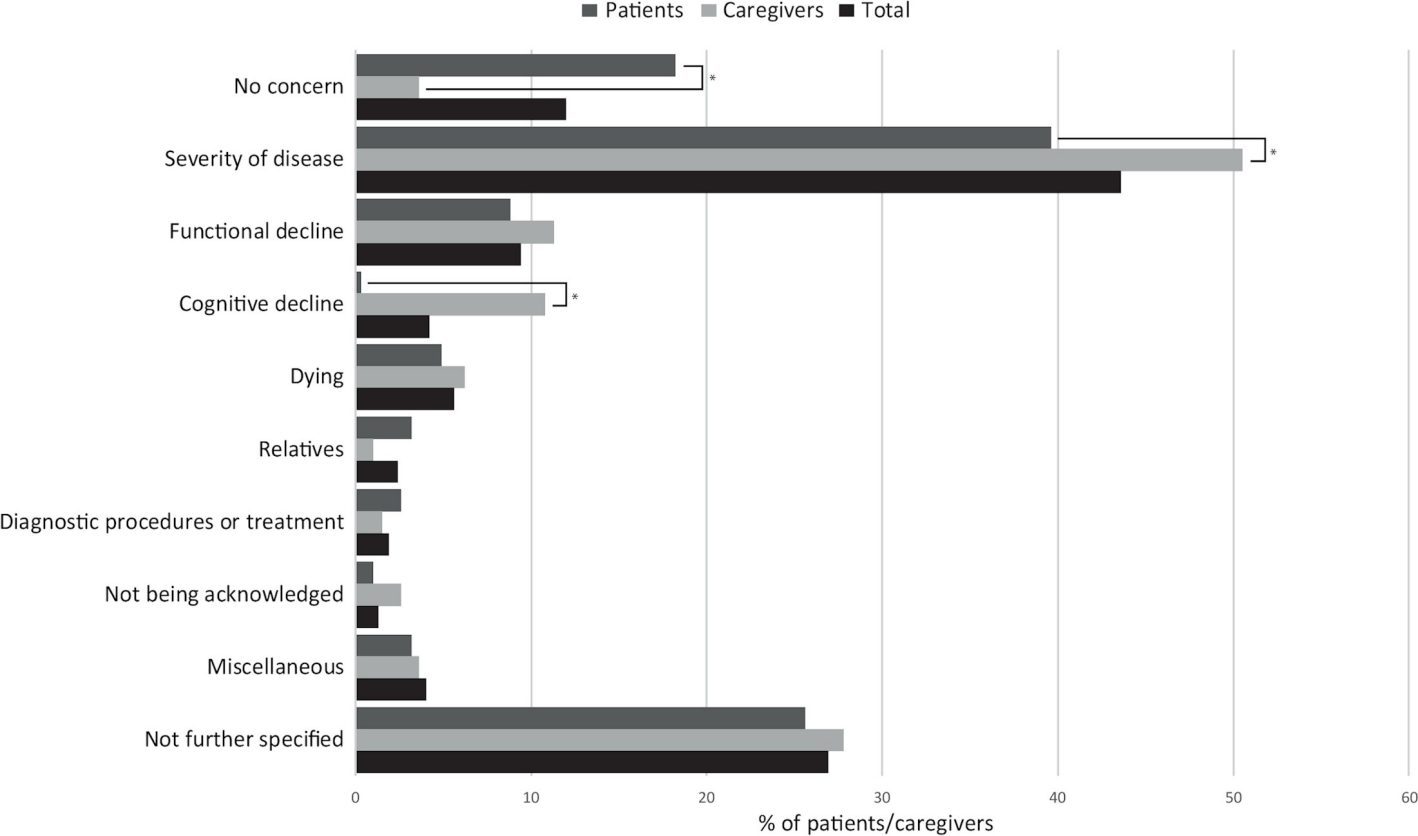
Concerns of patients and caregivers. This figure presents the type of illness related concerns of patients and caregivers in the first stage of the ED visit. Significant differences between patients and caregivers (p-value <0.05) are marked with an asterisk*.

The questionnaires were mostly answered by patients (51.9%), in 32.7% by the caregivers, while in 15.5%, it was unclear who filled out the form. A majority of respondents (n = 523, 88.0%) expressed at least one illness-related concern. In 363 (69.4%) of the questionnaires, the concerns were further specified. An illustration of the answers is presented in S1 File of S1 Table. Patients and caregivers were mostly concerned about the severity of disease (n = 259; 43.6%), functional decline (n = 56; 9.4%) and dying (n = 33; 5.6%; Fig 1).

Patients in who the respondent was concerned during the ED visit were younger (median 79 vs. 81 years, p-value 0.03) and their reason for the ED visit was more often because of cancer (9.4 vs. 1.4%) compared to patients in which the patient or caregiver was not concerned (S1 File of S2 Table).

### Differences in concerns between patients and caregivers and age groups

Patients were less often concerned about their medical condition than their caregivers (3.6 versus 18.2% resp., p <0.001, Fig 1). In addition, patients were less often concerned about the severity of their disease (39.6%) and about cognitive decline (0.3%) compared to the caregivers (50.5%, p = 0.016 and 10.8%, p<0.001, resp.).

When we compared the two age groups, we found no significant differences with respect to these items (Table 2).

**Table 2:** Concerns in different age groups^a^.

Type of concern	Age (years)	
	65–79 ≥80	
	(n = 284) (n = 239)	
No concern	31 (9.8)	40 (14.3)
Severity of disease	148 (47.0)	111 (39.8)
Functional decline	24 (7.6)	32 (11.5)
Cognitive decline	11 (3.5)	14 (5.0)
Dying	15 (4.8)	18 (6.5)
Relatives	9 (2.9)	5 (1.8)
Diagnostic procedures or treatment	5 (1.6)	6 (2.2)
Not being acknowledged	3 (1.0)	5 (1.8)
Miscellaneous	12 (3.8)	12 (4.3)
Not further specified	92 (29.2)	68 (24.4)

^a^No significant differences found between the age groups.

### Types of concerns and their association with outcome

We found that if a patient or caregiver was concerned about dying, this concern was associated with 30-day mortality (OR 2.89; 95%CI: 1.24–6.70, Table 3). Likewise, unspecified concerns were associated with mortality (OR 1.88; 95%CI: 1.09–3.22). In addition, the concern of dying was associated (OR 2.32; 95%CI: 1.12–4.82) with the secondary composite outcome (ICU/MCU admission, prolonged LOS, loss of independent living and unplanned readmission) as well. Concerns about functional or cognitive decline were not associated with the secondary loss of independent living outcome.

**Table 3:** Logistic regression analysis of type of concerns for different outcome measures.

Type of concern	Mortality		Composite endpoint^a^		Loss of independent living	
	OR (95% CI)	p-value	OR (95% CI)	p-value	OR (95% CI)	p-value
No concern (n = 71)	0.89 (0.39–2.05)	0.791	0.61 (0.36–1.03)	0.064	-	
Severity of disease (n = 259)	0.65 (0.38–1.12)	0.117	0.91 (0.66–1.27)	0.589	-	
Functional decline (n = 56)	-	-	-	-	1.08 (0.49–2.39)	0.852
Cognitive decline (n = 25)	-	-	-	-	2.11 (0.81–5.53)	0.127
Dying (n = 33)	2.89 (1.24–6.70)	0.014	2.32 (1.12–4.82)	0.023	-	
Not specified (n = 160)	1.88 (1.09–3.22)	0.022	0.98 (0.68–1.41)	0.915	-	

CI, confidence interval; OR, Odds ratio

^a^Composite endpoint: ICU/MCU admission, prolonged LOS, loss of independent living and unplanned readmission within 30 days

## Discussion

In this prospective multicenter study we showed that most older ED patients and their caregivers (88%) expressed a variety of concerns related to the patient’s medical condition or to the medical care, when they visit the ED. Patients and caregivers were particularly concerned about the severity of disease, functional decline and dying. Caregivers were more frequently concerned than patients, especially regarding the severity of disease and cognitive decline. Their concerns were also associated with adverse outcomes. This is illustrated by the finding that when there was a concern about dying, patients were almost three times more likely to die within 30 days.

To the best of our knowledge, our study is the first to explicitly ask older patients (or their caregivers) about their illness-related concerns during an ED visit using an open question. We found that most concerns were about the severity of disease, functional decline and dying. We focused on concerns upon arrival at the ED because it is possible that concerns dissipate after clarification of the medical problem and prognosis. To date, information regarding this topic is lacking. Most studies focused on the experience of patients during the ED visit (e.g. waiting times and communication by ED staff), and/or were performed in younger patients, smaller populations or other settings [4, 5, 7–9]. In only one Swedish study patients were explicitly interviewed about their concerns when visiting an outpatient clinic [6]. Their concerns were either practical or about their disease. Similar to our findings, they found that many patients could not specify the reason for their concern. A small Dutch qualitative study on the experience of patients during their ED stay [5] showed that most patients were concerned (144 concerns in 16 observed patients) and that many concerns were present early during the ED visit. However, in this Dutch study, patients were not explicitly asked about their concerns, and therefore, it is possible that not all concerns were disclosed. In addition, an American study [17], showed that many patients (25%) visiting the ED for suspected acute coronary syndrome were afraid to die. However, in this American study, patients answered closed questions in a questionnaire regarding several established concerns and therefore, it is possible that not all concerns were retrieved. Despite the differences in setting and design, our study and those of others show that most patients are concerned about their medical condition or need of medical care. Since patients often do not discuss their concerns with nurses or physicians [8, 10], they probably will not spontaneously discuss their concerns with researchers either. Therefore, it is likely that if patients (or caregivers) are explicitly asked about their concerns using an open question, they will reveal the (true nature of their) concerns, which in turn probably reflect their needs and preferences regarding medical care.

Interestingly, we found differences in concerns between patients and caregivers. Patients were less concerned with respect to disease severity or cognitive decline compared to their caregivers. The difference we found may be due to the nature or severity of disease for which the patients visited the ED, by the inability to assess the severity of their disease, by possible impairment in cognitive function or by the belief that one’s life is final or complete. In addition, it is possible that the questionnaires of patients with more severe underlying illness and/or cognitive impairment were more frequently filled out by caregivers. In line with our findings is that other studies show that the perception of a patients’ health differs between patients and caregivers [18–20]. Therefore, we think it is important to keep in mind that the concerns of patients and caregivers can be different as well as the needs of patients and caregivers during an ED visit.

Some of the concerns of patients and/or their caregivers were associated with adverse outcomes. When a patient/caregiver was concerned about dying, this concern was associated with 30-day mortality (OR 2.89) and the secondary composite outcome (OR 2.32). Moreover, unspecified concerns were associated with a higher risk of mortality (OR 1.88). This finding probably means that, for patients or caregivers, it is sometimes difficult to specify their concern of dying. Since many patients are concerned about dying, it may be important to ask our patients or caregivers about this concern. This could also provide an opportunity to talk about the patient’s goals, their preferences or end-of-life care, an important yet not often discussed topic in ED care [21, 22]. By discussing these subjects, one could avoid unwanted treatments and could improve quality of care.

Surprisingly, the concern about functional or cognitive decline was not associated with loss of independent living. This is in line with an UK study, showing that the expected need for additional care support by patients was not predictive of their actual need. An explanation for this finding is that in an early stage of the ED visit patients or caregivers are unaware of the nature and severity of the medical problem and its impact on the patient’s functioning. Another explanation might be that patients who are at risk of functional or cognitive decline are not aware of this. Since concerns of patients and caregivers are associated with adverse outcomes it is important to elucidate these concerns.

### Implications for clinical practice

We would like to address a few possible implications for clinical practice. First, we think it is very important to explicitly ask patients about their concerns when visiting the ED, since most patients are concerned but may be reserved to talk about these concerns to the staff. We think an open question is preferable because this offers an opportunity to freely communicate on all concerns. Second, because concerns are clearly associated with adverse outcomes, it is important to communicate with patients and caregivers about concerns regarding their prognosis and preferences regarding end-of-life care.

### Strengths, limitations and future perspectives

To date, this is the largest study regarding the concerns of older ED patients. Due to the inclusion of both patients and their caregivers a quite extensive study population could be analyzed, which strengthens our conclusion. A limitation of our study is that it was restricted to medical (internal medicine/gastroenterology) patients. It is not clear whether our results can be extrapolated to other patients visiting the ED. However, medical patients often present with complex medical problems, which are possibly accompanied by many different concerns. Another limitation is that we used questionnaires, while an open interview may have yielded more detailed information and could have explored other types of concerns. However, our study has shown that a qualitative study that explores the concerns of older ED patients is worth designing. In addition, as we did not control for confounders of adverse outcome we cannot conclude on whether the concerns of older patients are independently associated with the outcomes. This aspect of the concerns was not the scope of our study. Last, many patients or caregivers were concerned about losing independency. It is possible that the way societies take care of older patients influences this concern. In the Netherlands, if an older patient is in need of extensive homecare support, they will often move to a nursing home. In other cultures, in these circumstances, patients move in with their children, which may result in other kinds of concerns.

Future studies regarding this topic should focus on the concerns in the overall ED population, in other countries and use interviews in order to specify the types of concerns and explore the needs that ED patients, and especially older ED patients, have. These studies may provide insight in how to address and reduce these concerns and lead to more understanding and more empathic contacts with the health care workers in the ED. Other studies show that effective communication, pain management and involvement of caregivers helps reducing anxiety in ED patients [4, 8]. Therefore, the importance of early communication with patients about the nature of their medical problem, its’ impact on functioning and possible treatment options together with early involvement of caregivers in reducing concerns, anxiety and worries is another important subject to study.

## Conclusions

Older patients and particularly their caregivers are concerned about their medical condition or need for medical care when they visit the ED. These concerns are often justified since they are associated with serious adverse outcomes. We can easily clarify these concerns by asking them just one open question: ‘What are you concerned about?’. We think that it is important to find a moment to ask this question, because only when we are aware of the type of concerns or needs of our patients/caregivers, we are able to find ways to reduce these concerns and meet the needs in order to improve their experience and/or outcome and to make ED care more person-centered.

## Supporting information

S1 File Supplemental table. (DOCX)

S1 Dataset Dataset concerns. (SAV)
