# Supplementary material for: Concerns of older patients and their caregivers in the emergency department
Source: PLoS One. 2020 Jul 9;15(7):e0235708. doi: 10.1371/journal.pone.0235708 (PMC7347152; doi:10.1371/journal.pone.0235708)
Supplement: S1 File — (DOCX) [file pone.0235708.s001.docx]

**Supplemental Table**

**Concerns of older patients and their caregivers in the emergency department**

Noortje Zelis, MD, Sarah E Huisman, BSc, Arisja N Mauritz, MD, Jacqueline Buijs, MD PhD,

Peter W de Leeuw, MD PhD and Patricia M Stassen, MD PhD

**Table of contents**

Table S1…………………………………………………….p2

Table S2…………………………………………………….p4

**S1 Table. Concerns of patients and caregivers.***

| **Concerns** | **Total**  **N=363** |
| --- | --- |
| About (the severity of) the condition or illness | 32^a^ |
| Because of the uncertainty what causes the problems/illness | 29^a^ |
| About dying | 21^d^ |
| Because of the uncertainty regarding my condition | 20^a^ |
| About the (declining) health and functioning | 18^a,b^ |
| Because of the pain | 14^a^ |
| About not getting better | 12^a^ |
| About decline in cognitive and physical functioning | 10^b,c^ |
| Whether everything will turn out fine or not | 9^a^ |
| About cognitive problems or cognitive functioning | 9^c^ |
| Because he/she/I was never this ill before | 8^a^ |
| About age in relation to health condition or illness | 8^a^ |
| About having cancer | 8^a^ |
| Afraid that it is something serious | 8^a^ |
| About heart disease | 8^a^ |
| About losing independence and/or decline in physical functioning | 8^b^ |
| Uncertainty (about the future) | 8^h^ |
| Because this is the first ED visit and/or it all happened so quickly | 8^h^ |
| About shortness of breath | 7^a^ |
| About the test results | 6^a^ |
| About being unable to eat or drink and/or the risk for dehydration | 6^a^ |
| Because of the recurrent symptoms | 6^a^ |
| About severity of disease and cognitive functioning | 6^a,c^ |
| About the bleeding/ loss of blood | 5^a^ |
| About my (transplant) kidney(s) | 5^a^ |
| About losing a loved one | 5^d,e^ |
| About the high fever | 4^a^ |
| Because the therapy is not working | 4^a,f^ |
| About severity of disease and functional decline | 4^a,b^ |
| About quality of life | 4^b^ |
| About my loved ones | 4^e^ |
| About severity of disease and dying | 3^a,d^ |
| About loss of independence and afraid of dying | 3^b,d^ |
| About the Parkinson's disease | 3^a^ |
| Because it takes a long time (to recover) | 3^a^ |
| Afraid she cannot take care of her loved ones anymore | 3^b,e^ |
| Afraid of diagnostics or therapy (including surgery) | 3^f^ |
| About lung disease | 2^a^ |
| About being hospitalized for this illness | 2^a,f^ |
| Because my loved one is worried | 2^h^ |
| Because problems are getting worse and the patient cannot specify the symptoms or whishes | 2^a,g^ |
| Because the patient cannot accept the loss of independence | 2^b,g^ |
| About feeling miserable. About what needs to be arranged. | 1^a,b^ |
| About high blood pressure | 1^a^ |
| About my diabetes and COPD | 1^a^ |
| About osteoporosis | 1^a^ |
| About the mistakes and diseases in the past | 1^a,g^ |
| About the blood sugar being too low | 1^a^ |
| Afraid that I have something in my head | 1^a^ |
| Because she has a lot of medical problems | 1^a^ |
| Because the physician said she is pessimistic about the situation | 1^a^ |
| Because there's no treatment possible anymore | 1^a^ |
| I am a little bit worried | 1^a^ |
| I was frightened by the telephone call of the general practitioner | 1^a^ |
| I'm afraid something is wrong with my pancreas | 1^a^ |
| It is not that bad, it will be alright | 1^a^ |
| This medical problem was found by population screening | 1^a^ |
| Worried about cirrhosis of the liver | 1^a^ |
| Because she only recently started living on her own after her husband died | 1^b^ |
| Worried about work absence because of the severity of this illness | 1^a,b^ |
| Because he is not that old and still had around 10 years to enjoy life | 1^d^ |
| About the loss of my son 6 months ago | 1^e^ |
| Worried about real estate and relatives | 1^b,e^ |
| About the completion of chemotherapy | 1^f^ |
| Worried about this visit in relation to the chemotherapy | 1^f,h^ |
| Because it is unfortunate that my father cannot specify his wishes | 1^g^ |
| Whether I am being heard/believed | 1^g^ |
| Whether I am in good hands and not only seen by interns | 1^g^ |
| Because he is quickly worried about things | 1^h^ |
| Because my mom doesn't know how to continue | 1^h^ |
| About being desperate | 1^h^ |
| I am worried that something will happen at home | 2^h^ |

*Most of the concerns shown in the table are not the literal answers provided by patients or caregivers. Similar answers were combined in order to concise the table. The superscript characters ‘a’ to ‘h’ refer to the category in which we categorized the concerns: ^a^Severity of disease, ^b^Functional decline, ^c^Cognitive decline, ^d^Dying, ^e^Relatives, ^f^Diagnostic procedures or treatment, ^g^Not being acknowledged, ^h^Miscallaneous.

**Table S2. Overview of patients’ characteristics when a patient/caregiver was either concerned or was not concerned**

| **Patient characteristic** | **Patient/caregiver**  **Concerned**  **N=523** | **Patient/Caregiver**  **Not Concerned**  **N=71** |
| --- | --- | --- |
| Demographics |  |  |
| Median (IQR) Age, years | 79 (73-85) | 81 (75-86)^c^ |
| Male, n, % | 269 (51.4) | 40 (56.3) |
| Community dwelling, n (%) | 452 (86.4) | 63 (88.7) |
| Comorbidity and functional status |  |  |
| Median (IQR) CCI score | 2 (1-3) | 2 (1-3) |
| Median (IQR) Katz-ADL index score^a^ | 0 (0-2) | 0 (0-2) |
| Dementia, mild cognitive impairment or delirium, n (%)^b^ | 142 (28.6) | 22 (31.4) |
| Reason for ED-visit (ICD-10), n (%) |  |  |
| Infectious and parasitic disease | 157 (30.0) | 17 (23.9) |
| Diseases of the digestive system | 132 (25.2) | 23 (32.4) |
| Diseases of the circulatory system | 48 (9.2) | 6 (8.5) |
| Neoplasms | 49 (9.4) | 1 (1.4) |
| Endocrine, nutritional and metabolic diseases | 24 (4.6) | 7 (9.9) |
| Diseases of the respiratory system | 26 (5.0) | 4 (5.6) |
| Diseases of blood and blood-forming organs | 23 (4.4) | 4 (5.6) |
| Diseases of the genitourinary system | 24 (4.6) | 3 (4.2) |
| Miscellaneous | 40 (7.6) | 6 (8.5) |

SD, Standard Deviation; CCI, Charlson Comorbidity Index; ADL, Activities of Daily Living; ICD-10, International Classification of Diseases-10

^a^Katz-ADL index score determined in all hospitalized patients (n=471).

^b^Denominator count: n=496 and n=70, respectively

^c^significantly different with p-value <0.05
